# Workplace Stress, Presenteeism, Absenteeism, and Resilience Amongst University Staff and Students in the COVID-19 Lockdown

**DOI:** 10.3389/fpsyt.2020.588803

**Published:** 2020-11-27

**Authors:** Christina Maria Van Der Feltz-Cornelis, D. Varley, Victoria L. Allgar, Edwin de Beurs

**Affiliations:** ^1^Department of Health Sciences, Hull York Medical School, University of York, York, United Kingdom; ^2^Hull York Medical School, York, United Kingdom; ^3^Social and Behavioral Sciences, Psychology Department, Leiden University, Leiden, Netherlands

**Keywords:** workplace stress, study stress, COVID-19, presenteeism, absenteeism, mental health, vulnerability, resilience

## Abstract

**Background:** This study explored how the COVID-19 outbreak and arrangements such as remote working and furlough affect work or study stress levels and functioning in staff and students at the University of York, UK.

**Methods:** An invitation to participate in an online survey was sent to all University of York staff and students in May-June 2020. We measured stress levels [VAS-scale, Perceived Stress Questionnaire (PSQ)], mental health [anxiety (GAD-7), depression (PHQ-9)], physical health (PHQ-15, chronic medical conditions checklist), presenteeism, and absenteeism levels (iPCQ). We explored demographic and other characteristics as factors which may contribute to resilience and vulnerability for the impact of COVID-19 on stress.

**Results:** One thousand and fifty five staff and nine hundred and twenty five students completed the survey. Ninety-eight per cent of staff and seventy-eight per cent of students worked or studied remotely. 7% of staff and 10% of students reported sickness absence. 26% of staff and 40% of the students experienced presenteeism. 22–24% of staff reported clinical-level anxiety and depression scores, and 37.2 and 46.5% of students. Staff experienced high stress levels due to COVID-19 (66.2%, labeled vulnerable) and 33.8% experienced low stress levels (labeled resilient). Students were 71.7% resilient vs. 28.3% non-resilient. Predictors of vulnerability in staff were having children [OR = 2.23; CI (95) = 1.63–3.04] and social isolation [OR = 1.97; CI (95) = 1.39–2.79] and in students, being female [OR = 1.62; CI (95) = 1.14–2.28], having children [OR = 2.04; CI (95) = 1.11–3.72], and social isolation [OR = 1.78; CI (95) = 1.25–2.52]. Resilience was predicted by exercise in staff [OR = 0.83; CI (95) = 0.73–0.94] and in students [OR = 0.85; CI (95) = 0.75–0.97].

**Discussion:** University staff and students reported high psychological distress, presenteeism and absenteeism. However, 33.8% of staff and 71.7% of the students were resilient. Amongst others, female gender, having children, and having to self-isolate contributed to vulnerability. Exercise contributed to resilience.

**Conclusion:** Resilience occurred much more often in students than in staff, although psychological distress was much higher in students. This suggests that predictors of resilience may differ from psychological distress *per se*. Hence, interventions to improve resilience should not only address psychological distress but may also address other factors.

## Introduction

### Background

Since its onset in China in the fall of 2019, the worldwide outbreak of the COVID-19 pandemic has made a tremendous impact on people's lives, health, and livelihood. Many countries have put various levels of social restrictions in place. In the United Kingdom (UK), lockdown, social distancing and shielding of vulnerable people took effect from March 23, 2020. Non-essential shops and business were closed. People were advised to stay home except for essential trips for food, to the pharmacist, or the hospital. Only key workers, who performed essential tasks for society, including NHS employees, were allowed to go to their workplace. All other people were required work from home, if able. Many people lost their job temporarily or permanently and were on furlough, for which the Chancellor of the Exchequer installed a temporary scheme. Nurseries were closed, and children could not go to school, except children of key workers; and parents had to combine remote working with home-schooling and caring for their children.

Universities were closed as well, and examinations were canceled. Students were sent home from campus unless they had no place to go to, which was the case for international students who were unable to fly back home because all no-essential air travel was gradually stopped. These measures were put into effect in a relatively short time and lasted until June 15, 2020 when gradually non-essential shops could reopen again. By July 4, 2020, a further gradual easing of the lockdown started with the opening of pubs, restaurants and cinemas.

However, for University staff and students in the UK, the situation remained the same and laid bare how economically vulnerable the UK Universities were by their reliance on international, mostly Chinese students fees for income (1). The number of Chinese students coming to the UK each year has risen from 25,000 in 2006 to ~90,000 in 2019. Central funding of Universities by the government has dropped from 50% in 2010 to 25% in 2020 (2). As many Chinese students were confronted with hostility from UK residents in the wake of the COVID-19 outbreak (3, 4) with at least 267 offenses recorded in the first 3 months of 2020 (5), and due to the uncertainty on how teaching would commence again at the beginning of the new academic year, many Chinese students refrained from enrolling in UK Universities (6). Also, UK national students delayed starting their study by an anticipated 20% (7). This has affected job security of University teaching staff, and caused large changes in job content and, at some Universities, also payment for University staff (8).

Both for University staff and students, the rapidly changing work and study arrangements were deemed to cause work or study-related stress, which might be aggravated by personal stressors such as having to work remotely, having to change tasks, and having to combine all of this with home-schooling children and caring for shielding elderly family members or neighbors. This comes on top of having to deal with the general worries and anxieties emanating from the COVID-19 epidemic, deaths, and lack of testing available. Any symptoms that occurred and might be COVID-19 related could not be identified as COVID-19 for the first couple of months, which led to whole families having to self-isolate for 1–2 weeks if a family member had COVID-19 type symptoms. Further, people wondered when and how they would return to the University for work and study, and how they would deal with that. Psychological symptoms such as worries, physical symptom due to stress, especially stress due to remote working and living circumstances might lead to less work productivity related to the COVID-19 outbreak. This can be either sickness absence, termed absenteeism, or working with difficulty to do the tasks at hand, so-called presenteeism (9). Originally coined as “showing up at work while being sick” (10) because of chronic medical conditions (11) or because of work or personal characteristics (10), the emphasis in interpretation has shifted toward worker slowdowns in general and the economic costs associated with that (12). The prevalence of presenteeism is high, amounting to an average of 40% in a survey conducted amongst workers in 34 countries (13–15).

For some University staff and students it might be more difficult to deal with the crisis than for others, depending on factors affecting their resilience. Resilience being defined here as the ability to overcome adversity, which can be shown as experiencing no impact or positive impact on stress levels due to COVID-19, and functioning well in terms of work or study i.e., without presenteeism or absenteeism. From the literature, such factors might be age, ethnicity, living arrangements, job characteristics such as income level, educational background (16), physical fitness (17), psychological fitness, life experiences (18), personality and coping style (19–21). It may be that for some, the crisis brought some benefits as well. Some felt that no longer having to commute and being able to work from a relatively quiet workplace at home was less stressful than their regular working arrangements.

### Rationale

Hence, we felt a need to explore the impact of the COVID-19 pandemic on work stress levels and personal stress levels in University staff and students and investigate factors associated with resilience to meet the challenges of the COVID-19 crisis. We planned to explore work arrangements, work productivity and personal life, and mental and physical health and resilience, and investigate the influence of age, gender, living arrangements and ethnicity, such as Black, Asian, and minority ethnic (BAME) groups. BAME members of this group may be particularly vulnerable to COVID-19, more vulnerable to the impact of COVID-19-related regulatory measures, and may also have to deal with COVID-19 outbreak-related hostility in case of Chinese students or more general ethnicity related discrimination.

## Objectives

- To describe stress levels, mental health and physical health in University staff and students at the beginning of the imposed lockdown.
- To describe presenteeism and absenteeism and their association with the above.
- To investigate protective and vulnerability factors for the impact of the COVID-19 outbreak on work-stress and personal stress levels in staff and students. We will explore age, gender, ethnicity, childhood and current living arrangements, job characteristics, educational background, and chronic medical conditions.
- To explore predictors of resilience as the ability to overcome adversity, like experiencing no impact or positive impact on stress levels due to COVID-19, vs. a negative impact.

## Methods

This study followed a cross-sectional design. An online survey was sent to all University of York and Hull York Medical School (HYMS) staff and students. The survey was accessible via an anonymous link distributed via email to staff and students at the University of York and was announced by the Human Resources (HR) department and the student communications departments. The survey was open for 1 month from May 13, 2020 until June 22, 2020, and one email reminder was sent. Results are reported separately for students and staff.

### Variables

Variables are shown in Table 1.

**Table 1:** Variables.

**Variable**	**Assessment**	**Characteristics**
Current stress levels	Likert-type scale (22, 23)	Respondents rated their current work and personal stress levels using bespoke Likert-type scales.
Impact of COVID-19 on work and personal life stress	Likert-type scales (22, 23)	Respondents rated the impact of the COVID-19 pandemic on their stress levels using bespoke Likert-type scales. This included whether respondents felt the pandemic had a positive, negative, or no impact.
Perceived Stress	Perceived Stress Questionnaire (PSQ) (24)	A 30-item self-report questionnaire measuring perceived background stress during the past 2 years and circumstances known to provoke disease symptoms. Scores are summarized in a PSQ-Index ranging between 0 (lowest possible level of stress) and 1 (highest possible level of stress) Reliability (Cronbach's α) = 0.85 (25, 26).
Anxiety	Generalized Anxiety Disorder Screener (GAD-7)	The GAD-7 is a reliable 7-item self-report screening tool that measures the severity of anxiety and worry symptoms during the last 2 weeks. GAD-7 scores range from 0 to 21, and cut-off points of < 5, 5–10, and ≥ 10 represent normal, subclinical, and clinical levels of anxiety. Reliability (Cronbach's α) = 0.92 (27).
Depression	Patient Health Questionnaire (PHQ-9) (28)	The PHQ-9 is a reliable 9-item self-report questionnaire measuring the severity of depression during the past 2 weeks. Item scores ranged from 0 (not at all) to 3 (nearly every day), and total scores ranged from 0 to 27. Cut-off points of < 5, 5–10, and ≥ 10 represent normal, subclinical, and clinical levels of depression. Reliability (Cronbach's α) = 0.89 (28).
Somatic symptoms	Patient Health Questionnaire (PHQ-15)	The PHQ-15 is a reliable somatic symptom severity scale, consisting of a list of 15 somatic symptoms. Reliability (Cronbach's α) = 0.89 (29). In two studies in the occupational health setting in sick-listed employees, higher scores on the PHQ-15 were associated with more disability, longer sickness absence, and higher health-related job loss (30, 31). In a recent review of studies in primary care, the PHQ-15 was found to be equally effective or superior to other brief measures for assessing somatic symptoms and screening for somatoform disorders, with cut-off points of < 5, 5–10, and ≥ 10 represent normal, mild, and clinical symptom levels of physical symptoms (32).
Chronic medical conditions	CBS list (33)	A 31 item checklist for chronic medical conditions for which a patient received treatment from a doctor. Conditions are rated as somatic (i.e., known chronic medical conditions such as COPD and diabetes) or functional somatic syndromes (e.g., Irritable Bowel Syndrome (IBS), dizziness and back pain not explained by a known medical condition) Subscales are provided for the checklist.
Work absenteeism and presenteeism, job characteristics	iPCQ (34)	The *i*MTA Productivity Cost Questionnaire (*i*PCQ), is a short generic questionnaire assessing demographic and job characteristics (including education level and hours worked), presenteeism and absenteeism. The iPCQ applies to national and international studies for the measurement of productivity losses.
Job changes	Bespoke questionnaire	A bespoke questionnaire was developed which explored redundancy and furlough, and changes in work situation (such as remote working).
Resilience factors	Likert-type scales (22, 23)	A bespoke questionnaire containing 9 items exploring resilience using a Likert-type scale. Questions explored characteristics outlined in the literature (35) and focussed on access to outdoor space, and exercise levels, and childhood and current living environments.
Demographic questions	Bespoke questionnaire	A bespoke demographic questionnaire providing information on age, gender, ethnicity, nationality, educational level, work situation, relationship status, and living arrangements, including self-isolation.

#### Dependent Variables

- Stress experienced as measured by a VAS scale, psychological distress (PSQ, GAD-7, and PHQ-9), and absenteeism/presenteeism as in IPCQ are dependent variables.
- The impact of COVID-19 on personal and work stress levels as reported by respondents is taken as an indicator of resilience in this sample.

#### Predictors

- Age, gender, ethnicity, childhood, and current living arrangements, job characteristics, educational background, chronic medical conditions, personality, and stress reactivity style.

Physical symptoms and chronic medical conditions as known medical conditions and functional somatic syndromes.

### Analyses

Data is described descriptively using mean (sd) or n (%).

In order to establish the impact of COVID-19, mean scores of staff and students on screeners of psychological distress were compared to mean scores of normative samples using independent *t*-tests. The number of subjects with a healthy, subclinical, or clinical score on the dependent variables PHQ-9 and GAD-7 were established with normative values from pre-COVID-19 samples.

To investigate predictors of impact, we performed a hierarchical regression analysis with psychological distress (a composite score on the PSQ, PHQ-9, and GAD-7) as a dependent variable. For the binary dependent variables presenteeism and absenteeism, a logistic regression was performed using the same predictors that had been analyzed with psychological distress as dependent variable.

Subsequently, we divided the sample into two groups: subjects reporting a negative impact of COVID-19 on their stress levels (non-resilient), vs. subjects reporting a positive or neutral impact on their stress levels (resilient). Then an exploratory analysis was performed using regression logistic analysis to find additional vulnerability or protective factors for reported stress. All analyses were done for staff and students to explore if there are different predictors at play in both groups.

All analyses were performed on SPSS (v26). A *p-*value of < 0.05 was considered to indicate statistical significance.

### Other Analyses

We performed correlation analysis to explore associations between stress, mental health, presenteeism and absenteeism in the sample. We explored predictors of presenteeism and absenteeism, and we compared presenteeism and absenteeism in the resilient vs. the non-resilient subgroup.

We explored psychological distress scores and presenteeism and absenteeism scores in Chinese students compared to other Asian students.

We explored distress score differences between female, male, and non-binary genders and predictors of resilience in those gender categories.

## Results

### Description of the Sample

1,055 of 4,668 University staff (22.6%) and 925 of ~18,000 students (~5.1%) completed the survey.

#### Demographic Characteristics

Table 2 shows the demographic characteristics of the staff and student respondents. The mean age of staff was 45.2 years and 27.5 in students 74% of staff and students were female. Three staff members who responded to the survey were black, and 3% were Asian, vs. 3% black and 11% Asian in students. These demographic characteristics are representative of the wider UK University staff and student population (36–38).

**Table 2:** Demographic characteristics of the samples.

**Characteristics**		**Staff**		**Student**	
		***n***	**%**	***n***	**%**
Age		45.2 (30.5)		27.5 (31.8)	
Gender	Female	769	73	664	72
	Male	270	26	236	26
	Non-binary	8	1	21	2
Highest level of Education	I have never finished school or training programme	1	0	0	0
	Intermediate vocational secondary school	27	3	7	1
	Higher general secondary education	58	5	471	51
	School for higher vocational education	12	1	0	0
	University	880	83	418	45
	Other	76	7	25	3
Ethnicity	Asian	28	3	101	11
	Black	3	0	26	3
	White	987	94	746	81
	Other	33	3	50	5
Immigration Status	British/Dual Citizen	896	88	667	74
	Non-British/Dual Citizen	125	12	231	26
Chronic Medical Conditions	No CMC	637	60	634	69
	One CMC	247	23	181	20
	Multiple CMC	106	10	59	6
Chronic somatic medical conditions	No	758	71.8	734	79.5
	One	214	20.3	138	15.0
	Multiple	83	7.9	51	5.5
Childhood Environment	Rural Area	215	23	172	23
	Suburban area with access to parks/gardens/green areas	518	56	329	45
	Suburban area without access to parks/gardens/green areas	12	1	9	1
	Urban area with access to parks/gardens/green areas	124	13	152	21
	Urban area without access to parks/gardens/green areas	10	1	15	2
	A mix of the above	54	6	58	8

A third of staff respondents (33%) and a quarter of students (26%) reported at least one chronic medical condition. A high proportion of staff (92%) and students (89%) had access to green spaces where they lived in childhood.

#### COVID-19 Related Work and Living Characteristics

The COVID-19 related work and living characteristics are presented in Table 3.

**Table 3:** COVID-19 related work and living characteristics in staff and students.

**COVID-19 related work and living characteristics**		**Staff**		**Student**	
		***n***	**%**	***n***	**%**
Have to work from home because of the COVID-19 situation	Yes	913	98	566	78
	No	22	2	164	22
Lost their job because of the COVID-19 situation	Yes	6	1	51	7
	No	923	99	675	93
Missed work in the last 4 weeks as a result of being sick	No	970	93	318	90
	Yes	70	7	34	10
During the last 4 weeks there were days in which they worked but during this time were bothered by physical or psychological problems	No	403	39	186	52
	Yes	636	61	169	48
In social isolation since the outbreak (e.g., due to a suspected COVID-19 infection or because you are at risk of infection)?	Yes	244	26	291	40
	No	687	74	444	60
Children/step-children living with them	Yes	428	41	89	10
	No	624	59	834	90
	Prefer not to say	3	0	2	0
Current Environment	Rural Area	185	20	136	18
	Suburban area with access to parks/gardens/green areas	505	54	323	44
	Suburban area without access to parks/gardens/ green areas	8	1	13	2
	Urban area with access to parks/ gardens/green areas	212	23	236	32
	Urban area without access to parks/gardens/green areas	14	2	10	1
	A mix of the above	8	1	18	2
Do you have access to an outdoor space at home?	Yes, to a garden	716	76	521	70
	Yes, to a courtyard	117	13	85	11
	Yes, to a balcony	33	4	45	6
	No	70	7	91	12
Compared to the time before COVID-19 social distancing measures were put in place, how much exercise are you currently doing?	A lot less exercise	196	21	210	28
	Somewhat less exercise	230	25	176	24
	About the same amount	240	26	151	20
	Somewhat more exercise	215	23	142	19
	A lot more exercise	55	6	63	8

A high proportion of the staff (98%) and students (78%) surveyed had their work and study arrangements changed and were working or studying remotely because of the COVID-19 pandemic. 1% of staff and 7% of students lost their job or dropped out of their study. 7% of staff and 10% of students were sick-listed in the last 4 weeks.

A quarter of staff (26%) and 40% of students had experienced problems doing their work or studying because of psychological or physical symptoms (presenteeism).

Regarding living arrangements, 26% of staff and 40% of students were in social isolation due to the COVID-19 outbreak. 41% of staff and 10% of students had children living in with them. Most had access to green space (e.g., a garden). However, 7% of staff and 12% of students had no direct access to a garden or balcony in their home during the lockdown. Participants were asked whether they were exercising more, less or at the same level as they were before the lockdown was put in place. Exercise levels since lockdown were evenly distributed.

### Impact on Stress and Mental Health

#### Psychological Distress

The mean PSQ scores were 0.51 (±0.2), in students (*n* = 788) and 0.43 (±0.2), in staff (*n* = 965). Regarding the VAS score indicating the level of personal stress, 79% of staff who completed this question reported elevated stress levels due to COVID-19. 66% reported that COVID-19 raised their work stress level.

Almost a quarter of staff (22.1%) reporting on the GAD-7 (*n* = 965) had anxiety scores indicating probable anxiety disorder (GAD-7 score ≥ 10), and 24% reported depression scores indicating probable depressive disorder (PHQ-9 score ≥ 10), whereas students reported 37.2 and 46.5%.

Proportions of non-clinical, subclinical, and clinical levels of distress are shown in Table 4.

**Table 4:** Proportions of non-clinical, subclinical, and clinical levels for the three dependent variables measuring distress in the present sample.

	**Staff**				
	**Mean**	**SD**	**0–4** **Non-clinical**	**5–9** **subclinical**	**10 or higher** **clinical level**
Anxiety	6.41	5.36	43.4	34.6	22.1
Depression	6.11	4.93	44.2	31.8	24.0
			**0–0.33** **Non-clinical**	**0.34–0.45** **subclinical**	**0.46–1** **clinical level**
Stress	0.43	0.19	33.3	20.0	46.7
	**Students**				
	**Mean**	**SD**	**0–4**	**5–9**	**10 or higher**
Anxiety	8.31	5.74	28.8	34.0	37.2
Depression	9.87	6.57	25.3	28.2	46.5
			**0–0.33** **Non-clinical**	**0.34–0.45** **subclinical**	**0.46–1** **clinical level**
Stress	0.51	0.20	19.4	19.1	61.5

**Diagram 1:**
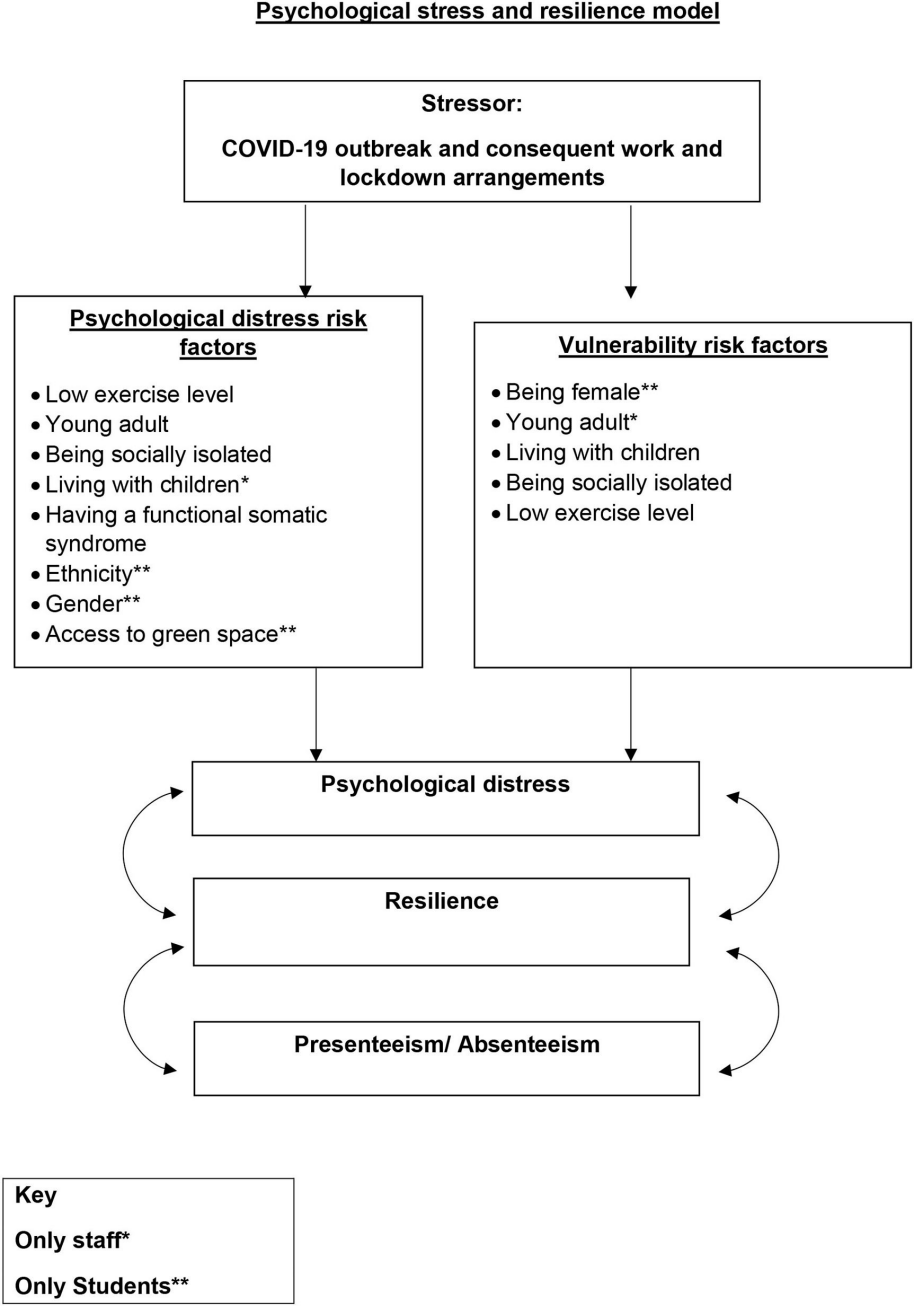
Psychological stress and resilience model.

Diagram 1 (below) details the impact of stress and resilience factors on psychological distress.

#### Experienced Stress Levels

##### Staff

**Figure 1:**
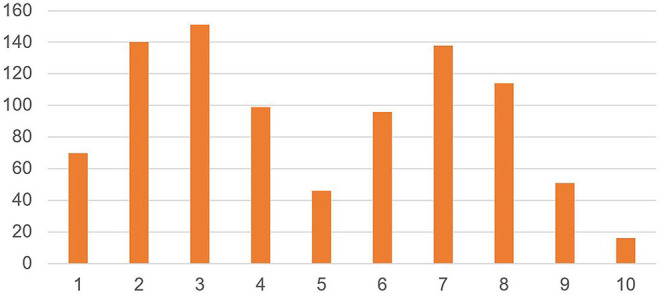
Experienced stress level in staff (*N* = 921). On a scale of 1–10, where 1 is no personal stress, and 10 is considerable personal stress, how would you score the level of your current personal stress?

Figure 1 shows experienced stress levels in staff as indicated by the VAS score and reveal a bimodal distribution of scores, with most staff either experiencing high stress levels and or low stress levels and few scoring in between [Mean = 4.9 (±2.5)].

##### Students

Regarding the VAS score indicating the level of personal stress over the past 2 weeks, 72% of the students who completed this question reported elevated stress levels due to COVID-19. 70% reported that COVID-19 raised their study stress levels.

**Figure 2:**
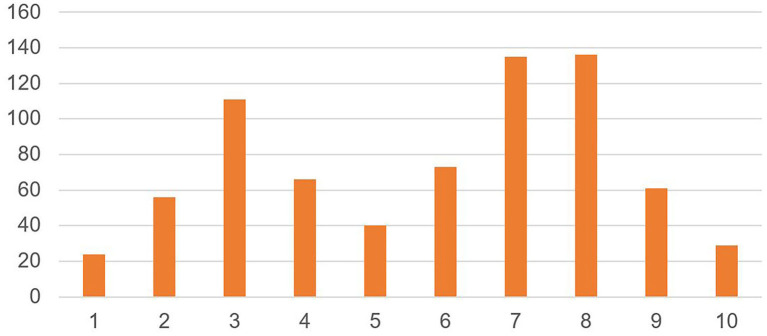
Experienced stress level in students (*N* = 731). On a scale of 1–10, where 1 is no personal stress, and 10 is considerable personal stress, how would you score the level of your current personal stress?

Figure 2 shows experienced stress levels in the students, as indicated by the VAS score and as with staff, is a bimodal distribution [mean = 5.8 (±2.5)].

#### Composite Psychological Distress Score

Correlations among scores on the GAD-7, PHQ-9, and PSQ were high: r_GAD−PHQ_ = 0.78 for staff (0.77 for students); r_GAD_PSQ_ 0.73 (0.71 for students); r_PHQ_PSQ_ = 0.71 (0.73 for students). With PSS they were somewhat lower); r_GAD_PSS_ 0.58 (0.58 for students); r_PHQ_PSS_ = 0.52 (0.52 for students).

These correlations suggest that the measures assess highly similar constructs and support the construction of a composite measure of the GAD-7, PHQ-9, and PSQ, reflecting an overall level of psychological distress (depression, anxiety, and perceived stress). Thus, scores on the PHQ-9, GAD-7, and PSQ were standardized and combined into a composite score for psychological distress.

### Predictors of Psychological Distress

Psychological distress is a condition where a person feels emotional suffering (including feeling anxious, scared, tired, or sadness) due to stressors. Stressors may include health issues, everyday stressors (such as work or personal stress) or traumatic experiences. With multiple regression analysis, we examined separately for staff and students, which predictor variables (listed in Table 1) were significantly associated with psychological distress as the dependent variable. Stepwise, forward entry resulted in a model comprising a set of five variables that significantly predicted psychological distress in staff: a lessened current exercise level (β = −0.23), lower age (β = −0.24), reporting social isolation (β = −0.13), more functional somatic syndromes (β = 0.14), and having (step)children living at home (β = −0.09), are associated with more distress. The variables combined were associated (*r* = 0.40) with psychological distress, explaining 15.9% of the variance in psychological distress of staff members.

For students, a set of seven variables predicted distress: lower age (β = −0.20), reporting social isolation (β = −0.17), not being of Asian descent (β = −0.11), female gender (β = −0.11), a lessened current exercise level (β = −0.10), living in an urban environment (β = −0.09), and more functional somatic syndromes (β = −0.08) were associated with more psychological distress. The combination of variables was associated (*r* = 0.34) with distress, explaining 11.3% of the variance in distress of students. The results are shown in Table 5.

**Table 5:** Bivariate correlations between predictors and psychological distress and standardized β's for predictors in a model resulting from multiple regression analyses for staff and students.

		**Staff**				**Students**			
**Predictor**	**Range**	**M**	**SD**	**Bivariate r**	**Stand. β**	**M**	**SD**	**Bivariate r**	**Stand. β**
Age	18–81	44.3	11.4	−0.23^***^	−0.24	26.5	9.6	−0.19	−0.20
Childhood environment	1–5	2.09	0.96	−0.07^*^		2.27	1.14	0.05	
Current environment (urban)	1–5	2.31	1.08	0.05		2.53	1.17	0.05	−0.09
Exercise level (lower)	1–5	2.68	1.20	0.23^***^	−0.23	2.56	1.31	0.10^**^	−0.10
Outdoor space	1–4	1.42	0.87	0.06		1.60	1.05	0.04	
		**N**	**%**			**N**	**%**		
Gender	Male	270	36.0	−0.01		236	26.2	−0.10	−0.11
Education	1/2	1	0.1	0.06		7	0.8	−0.11^**^	
	3					471	52.6		
	5	27	2.6			418	46.7		
	6	58	5.5						
	7	12	1.1						
	8	880	83.4						
White		987	93.6	−0.02		746	80.8	0.06	
Black		3	0.3	0.03		26	2.8	−0.07^*^	
Asian		28	2.7	0.04		101	10.9	−0.07	−0.13
Other		33	3.1	−0.01		50	5.4	−0.06	
Immigration status	British	896	84.9	0.03				−0.06	
Having Children	YES	428	40.7	0.04	−0.09	89	9.6	0.08^*^	
IPCQ4 (absenteeism)	YES	70	6.7	−0.04		34	9.7	−0.01	
IPCQ7 (presenteeism)	Yes	636	61.2	0.04		169	47.6	−0.05	
CMC-somatic	None	758	71.8	0.08^*^		734	79.4	0.02	
CMC-functional	None	956	90.6	0.16^***^	0.14	867	93.9	0.09^**^	−0.08
Social Isolation	Yes	244	26.2	0.19	−0.13	291	39.6	0.14^***^	−0.17

* p < 0.05;

** p < 0.01;

*** *p < 0.001*.

We explored further how being a student of Asian descent seemed associated with lower psychological distress by comparing mean PSQ, GAD-7, and PHQ-9 scores between Chinese and non-Chinese Asian students. Forty-seven of the 101 Asian students were Chinese, the other ones were from a large variety of Asian countries (such as Japan, Korea, Singapore, Thailand and Vietnam). The Chinese students had lower scores on the GAD-7 (6.25 ± 4.89) and the PHQ-9 (7.95 ± 6.05) compared to the other Asian students (7.98 ± 6.23 and 9.86 ± 7.37), although this was not a significant difference.

### Predictors of Resilience to COVID-19 Related Psychological Distress

A bespoke question was developed to measure the extent of impact of COVID-19 on stress levels. Participants were asked to rate the extent of the impact on a 5 point scale (−2 considerable negative impact to +2 considerable positive impact, with 0 indicating no impact). We created two subgroups: people reporting elevated stress levels due to COVID-19 were coded in the non-resilient, or vulnerable, group. People reporting no impact or positive impact of COVID-19 on their stress levels were coded in the resilient group. This division was based on the logic that resilience would mean the ability to be not impacted by the COVID19 outbreak, or even positively impacted, as resilience is considered the ability to deal with stress and overcome it (16, 18, 21). As stated by Miller-Lewis et al. (39), a gold standard benchmark has not yet been established to operationalise resilience. Although there are a number of ways to operationalise resilience, a binary approach was chosen for this study as this work will involve multiple follow-up data waves collecting a wide range of continuous data, and data-driven methods are more suitable for this process than definition-driven methods (40).

In staff, 565 (66.2%) were non-resilient and 288 (33.8%) were resilient. In students, 485 (71.7%) were resilient vs. *N* = 191 (28.3%) non-resilient. A logistic regression analysis was conducted to explore predictors of vulnerability/resilience. For staff, a model of four variables predicted vulnerability [c^2^(6) = 5.56; *p* < 0.001]. Respondents with high vulnerability are younger, have children, report social isolation, and report a low current exercise level. For students, a similar model of four variables predicted vulnerability [c^2^(3) = 32.14; *p* < 0.001]. Being female, having children, social isolation, and a low current exercise level was associated with higher vulnerability. The results are shown in Table 6.

**Table 6:** Results of logistic regression analyses of predictors for vulnerability, presenteeism, and absenteeism among staff and students.

	**Vulnerability**		**Presenteeism**		**Absenteeism**	
**Predictor**	**OR**	**CI (95)**	**OR**	**CI (95)**	**OR**	**CI (95)**
**STAFF**						
Age	0.98	0.97–0.99	0.97	0.96–0.98	0.97	0.96–0.98
Gender (male)						
Education						
Childhood environment						
White						
Black						
Asian						
Other						
Immigration status						
Having Children	2.23	1.63–3.04				
Current environment (urban)						
Exercise level	0.83	0.73–0.94	0.78	0.69–0.89		
Outdoor space			1.26	1.04–1.55		
IPCQ4 (absenteeism)						
IPCQ7 (presenteeism)						
CMC—somatic			1.34	1.03–1.74	1.53	1.01–2.28
CMC—functional			2.14	1.21–3.80	2.02	1.10–3.71
Social Isolation	1.97	1.39–2.79	1.53	1.05–2.23	2.62	1.48–4.63
**STUDENTS**						
Age						
Gender (male)	1.62	1.14–2.28				
Education			2.02	1.52–2.69		
Childhood environment			0.73	0.57–0.95		
White						
Black						
Asian			5.03	1.55–16.29		
Other						
Immigration status						
Having Children	2.04	1.11–3.72				
Current environment (urban)						
Exercise level	0.85	0.75–0.97			0.62	0.39–0.97
Outdoor space						
IPCQ4 (absenteeism)						
IPCQ7 (presenteeism)						
CMC—somatic						
CMC—functional					4.19	1.50–11.69
Social Isolation	1.78	1.25–2.52			2.99	1.09–8.23

For staff, a model of four variables predicted vulnerability [χ^2^(6) = 5.56; *p* < 0.001]. Respondents with high stress are younger [OR = 0.98; CI (95) = 0.97–0.99], have children [OR = 2.23; CI (95) = 1.63–3.04], report social isolation [OR = 1.97; CI (95) = 1.39–2.79], and report a low current exercise level [OR = 0.83; CI (95) = 0.73–0.94].

For students, a model of four variables predicted vulnerability [χ^2^(3) = 32.14; *p* < 0.001]. Respondents with higher stress are females [OR = 1.62; CI (95) = 1.14–2.28], have children [OR = 2.04; CI (95) = 1.11–3.72], report social isolation [OR = 1.78; CI (95) = 1.25–2.52], and a low current exercise level [OR = 0.85; CI (95) = 0.75–0.97].

### Presenteeism and Absenteeism

#### Association of Presenteeism and Absenteeism With Psychological Distress

We examined the association between absenteeism and presenteeism and the dependent variables PHQ-9, GAD-7, and PSQ, and their composite. The correlation between psychological distress and presenteeism (0.435) was much higher than the correlation between psychological distress and absenteeism (0.133).

#### Predictors of Presenteeism and Absenteeism

We performed a logistic regression analysis to explore the predictors of presenteeism and absenteeism.

For staff, a model of six variables predicted presenteeism [χ^2^(6) = 68.40; *p* < 0.001]. Predictors of presenteeism are younger age [OR = 0.97; CI (95) = 0.96–0.98], living with a somatic chronic medical condition [OR = 1.34; CI (95) = 1.03–1.74] or a functional somatic syndrome [OR = 2.14; CI (95) = 1.21–3.80], social isolation [OR = 1.53; CI (95) = 1.05–2.23], no access to outdoor space at home [OR = 1.26; CI (95) = 1.04–1.55], and low current exercise level [OR = 0.78; CI (95) = 0.69–0.89].

For students, a model of three variables explained presenteeism [χ^2^(3) = 36.38; *p* < 0.001]. Predictors of presenteeism are education level [OR = 2.02; CI (95) = 1.52–2.69], being of Asian ethnicity [OR = 5.03; CI (95) = 1.55–16.29] and childhood environment without access to green spaces [OR = 0.73; CI (95) = 0.57–0.95].

Among staff, a model comprising four variables explained absenteeism [χ^2^(4) = 29.80, *p* < 0.000]: lower age [OR = 0.97; CI (95) = 0.95–0.99], living with a somatic chronic medical condition [OR = 1.53; CI (95) = 1.01–2.28], with a functional somatic syndrome [OR = 2.02; CI (95) = 1.10–3.71] and living in social isolation [OR = 2.62; CI (95) = 1.48–4.63].

Among students a model of three variables predicted absenteeism [χ^2^(3) 17.27, p = 0.001]: the presence of functional somatic syndromes [OR = 4.19; CI (95) = 1.50–11.69], living in social Isolation [OR = 2.99; CI (95) = 1.09–8.22], and exercise level [OR = 0.62; CI (95) = 0.34–0.97].

#### Predictors of Presenteeism and Absenteeism in Resilient or Non-resilient Staff and Students

We created two subgroups: People reporting a negative impact of COVID-19 on their stress levels were coded in the non-resilient group. People reporting no impact or positive impact of COVID-19 on their stress levels were coded in the resilient group.

Presenteeism was significantly lower in resilient staff (*p* < 0.001), but there was no significant difference for absenteeism. None of the factors for students were statistically significant.

### Gender

A separate analysis explored psychological distress according to three gender categories. Males (N = 437) reported the lowest distress score (M = −0.08 ± 0.93) of all gender categories. Females (*N* = 1,251) had higher scores (M = 0.03 ± 0.90) but the non-binary gender group (*N* = 36) had the highest distress score (M = 0.31 ± 0.94). However, the non-binary gender group did not differ to a statistically significant degree from the others.

We explored if gender was associated with resilience, including non-binary gender. Although the percentages for non-binary gender seem to hint to less resilience than males and females, there were no significant differences between gender categories in staff (*p* = 0.272) or students (*p* = 0.635).

We also explored if presenteeism and absenteeism were associated with gender and found no significant differences for staff or students.

### Age

We explored if participant age was associated with psychological stress. In the analysis, participants were separated into two groups; aged under 30 years and aged 30 years and above. This is in conjunction with Levinson's (41) theory of adult development stating that the first age of early adulthood is between 28 and 30. It was found that younger adults (aged under 30) were more likely to be suffering from psychological distress (41).

There was a significant difference in the PHQ-9 between younger (M = 7.69, SD = 5.271) and older (M = 6.33, SD = 5.360) staff members; t_(936)_ 2.415, *p* = 0.016. A significant difference was also found in younger (M = 7.18, SD = 5.008) and older (M = 6.03, SD = 4.907) staff members in the GAD-7; t_(949)_ 2.252, *p* = 0.025. However, no significant difference was found for the PSQ in younger (M = 0.4196, SD = 0.17959) and older (M = 0.4356, SD = 0.19800); t_(949)_ −0.791, *p* = 0.429.

Similar to the staff members, there was a significant difference in the PHQ-9 between younger (M = 10.55, SD = 6.42) and older (M = 7.92, SD = 6.636) students; t_(747)_ 4.782, *p* = 0.00. A significant difference was also found in younger (M = 8.77, SD = 5.789) and older (M = 6.84, SD = 5.288) students in the GAD-7; t_(772)_ 4.235, *p* = 0.00. For the PSQ, a significant difference was also found between younger (M = 0.5165, SD = 0.18928) and older students (M = 0.4753, SD = 0.21482); t_(772)_ 2.347, *p* = 0.020.

## Discussion

This study found that University staff and students were severely affected by the COVID-19 outbreak, the change in work and study arrangements and the lockdown. A high proportion of the staff (98%) and students (78%) surveyed worked or studied remotely because of the COVID-19 outbreak. 1% of staff and 7% of students lost their job or dropped out of their study. 7% of staff and 10% of students reported sickness absence, and 26% of staff and 40% of the students experienced presenteeism in the last 4 weeks.

### Psychological Distress

The mean anxiety levels in staff respondents are twice the mean score than in an *N* = 5,030 general population study in Germany reported by Lowe et al. (42) and students score almost three times higher. General population levels in a USA study (43) are higher than the German levels. Nevertheless, they are still substantially lower than the anxiety levels found in our samples. For both staff and students, these means differ significantly from the German and US norms. For example, for staff the difference with the German mean (Mdiff = 3.14; t_(5, 993)_ = 24.32; *p* < 0.0001) and for students (Mdiff = 5.34; t_(5, 816)_ = 36.81; *p* < 0.0001).

For depression, the mean PHQ-9 scores in our sample are similarly higher compared to a German (*N* = 5,018) general population study (44), with staff scoring twice as high and students more than three times higher. For example, for staff the difference with the German mean (Mdiff = 3.51; t_(5, 981)_ = 25.86; *p* < 0.0001) and for students (Mdiff = 6.97; t_(5, 804)_ = 44.86; *p* < 0.0001). University staff scored higher compared to scores of 1,242 Chinese residents of the Wuhan province collected in the second half of February 2020; among our staff, 56.7% had symptoms of anxiety (GAD-7, ≥5) compared to 27.5% in the Chinese sample. 55.8% had symptoms of depression vs. 29.3% of the Chinese (PHQ-9, ≥5) (45).

British students scored only slightly lower than 340 Brazilian medical students during the COVID-19 epidemic. Their average GAD-7 mean score was 9.18 (± 4.75), and their average PHQ-9 mean score was 12.72 (± 6.62) (46).

Our percentages for clinical caseness for staff coincide well with recent findings during COVID-19 in Austria, where 21.0% scored above the cut off ≥10 points (PHQ-9) and 19.0% scored above the cut-off ≥10 points (GAD-7) for moderate anxiety symptoms (47). The percentage of students in our sample scoring in the clinical range is much higher (37.2% with anxiety and 46.5% with depression). These are concerning percentages as, due to financial constraints, treatment provision, especially for students, is limited.

Regarding the stress scores on the PSQ, based on a Swedish population sample Bergdahl and Bergdahl (48) recommend a score of 0.34 or higher as indicating moderate perceived stress and 0.46 or higher as a high level of perceived stress. 46.5% of the staff and 61.5% of the students score 0.46 or higher. To summarize, the respondents in our sample were substantially affected by the COVID-19 crisis and the measures taken to contain the spread of the virus.

### Presenteeism and Absenteeism

Stress levels, anxiety and depression, are correlated and are associated with presenteeism and absenteeism. The correlation between psychological distress and presenteeism (0.435) was much higher than the correlation between psychological distress and absenteeism (0.133) though, suggesting that the drivers for absenteeism may be less related to psychological distress than the drivers for presenteeism.

Presenteeism is high in both groups, and the percentages of absenteeism are much higher than usual in the educational sector. Students are more afflicted than staff, and this may well hang together with their younger age (49), and their being in a transitional phase as adolescents moving from the safe environment of the parental home to a non-permanent residence at University campus to build new networks and obtain grades in order to secure a job in the future. Many students self-fund their study so the insecurity around the suspension of study activities and the economic insecurity may have more influence on them than on staff.

If we look at predictors of presenteeism and absenteeism in staff, we find that young age is a factor. However, the effect size for young age was minimal, with an OR of 0.97, and hence of limited relevance. Factors with higher effect sizes predicting presenteeism in staff were living with a physical chronic medical condition or a functional somatic syndrome, social isolation, having no access to outdoor space at home, and low exercise level. Most of these also are predictors of psychological distress. It might be that the combination of having a chronic medical condition, no access to outdoor space at home and limited exercise options during the lockdown, may have contributed to more physical symptoms and presenteeism. For students, predictors of presenteeism are education level, Asian ethnicity and lack of access to green space in the childhood environment. York, UK (where the University of York is situated) has a wealth of green and blue space, including multiple nature reserves, parks, rivers and lakes. In addition, the area is in close proximity to the Yorkshire Dales, Yorkshire Moors and multiple seaside areas. It can be assumed that a high number of staff and some students taking part in the study live in York and have access to these green and blue spaces. This finding aligns with a study confirming the relevance of long-term exposure to greenery to resilience, although having access to work had more effect on resilience (50).

We found it remarkable that Asian students were much more vulnerable to presenteeism, with an OR of 5, but less prone to report psychological distress. In our study, 46.5% of the Asian students were Chinese, and the remainder came from a variety of countries in Asia. This finding might imply that Chinese students in case of stress may report lower on psychological distress, but experience their stress more in terms of presenteeism. The literature suggests that there may be cultural differences in how Chinese people communicate distress, compared to, for example, people from western culture. Chinese people have been suggested to report physical symptoms rather than psychological symptoms such as depression (51, 52) and anxiety (53), and this tendency might originate from the way people showing psychological distress were treated during the Cultural Revolution (54, 55). Also, more in general, stigma related to mental disorders might play a role in the tendency to under-report psychological distress (56). In such circumstances, presenteeism might be a choice of the individual to deal with psychological distress by working (9), although that was found more difficult to do than normally. Such a mechanism has been proposed in a study in Chinese workers in Japan (57), and it might play a role here as well. In that particular study, an intervention to promote a health-related lifestyle showed good results in terms of presenteeism, work-related stress, and mental health.

### Resilience

It is noteworthy that resilience occurred much more often in students than in staff, whereas psychological distress was much higher in students. This suggests that predictors of resilience may differ from psychological distress *per se*. In other words, a person may feel psychological distress and nevertheless be resilient. Hence, interventions to improve resilience should not only address psychological distress but may also address different factors that contribute to resilience, or aim at improving skills to deal with stressors.

The bimodal distribution of the experienced stress levels due to COVID-19 that occurred in both staff and students allowed us to explore vulnerability and resilience factors in both. We found that younger age, lack of exercise, social isolation and having to take care of children while remote working from home predict higher distress levels in staff, and so does social isolation. Also, having functional somatic syndromes is associated. Young age can contribute to vulnerability to psychological distress, and in this particular setting, staff with young age may have less job security as they are more often academics on a temporary contract than older staff. The positive effect of exercise on anxiety and depression levels has been reported widely (58, 59) and was confirmed in this study. The finding that functional somatic symptoms are predictors of psychological distress supports their often being conceived as stress-related symptoms (60, 61). In this sample, they might either be a somatic expression of the high experienced stress levels, or an indicator of a pre-existing stress-related condition as a trait marker for longstanding high stress levels that increase vulnerability. The total variance explained by these predictors taken together amounts to a moderate effect size (62), which is substantial.

### Gender

For students, predictors of vulnerability were identifying as female, having children, reporting social isolation, and a low current exercise level and the variance explained also amounts to a medium effect size. Having to look after one's children that were not allowed to go to the nursery or school because of lockdown, obviously would be a significant impediment for trying to study from home and one wonders if the childcare-related tasks might have befallen mostly on female students, possibly having to support the father of the household to work for the family income remotely. This is supported by a recent study conducted by Carroll et al. (63) who explored the impact of COVID-19 on health behaviors and stress, and found that mothers reported higher stress levels (mean 6.8) than fathers (mean 6.0) and mothers reported a greater decrease in current exercise level (59%) than fathers (52%). That might be a gender-related vulnerability factor.

Regarding gender, we found no significant differences between males, females and non-binary gender regarding psychological distress or resilience. The non-binary gender group had the highest distress score and seemed less resilient, but this was not statistically significant. However, that might have to do with their low number.

### Limitations of the Study

This is a study based upon a survey amongst staff and students of the University of York. All staff and students received an invitation to participate in the survey, several announcements, and a reminder. 22.5% of the staff responded, and probably this study can be considered representative in terms of the staff. However, only ~5.1% of the students responded, so there will be an unknown amount of selection bias and representiveness, especially regarding the students. All students were sent home in March 2020 and possibly had limited access to email, especially if they came from lower socioeconomic areas. In addition, students may not have had access to technology or adequate internet access to be able to complete the survey. One could also argue that people who also had caring commitments or were too stressed and overburdened would not fill in the survey; on the other hand, it might as well be that persons who felt well did not feel the need to fill in the survey. With a low response rate, respondents with a more extreme attitude may be overrepresented as having strong feelings about the subject matter will stimulate responding. The generalisability is therefore limited to an unknown degree, and this is the main limitation of this kind of surveys (64).

Regarding the representativeness of the student sample, we compared our sample to recent UK University staff and student demographic statistics. In 2017/18, there were 429,560 UK University employees, with 49% filling academic and research roles (36). One in five University staff members are international, with ~60% coming from an EU country (38). This is representative of our sample, with 45% of respondents in academic and research roles. Nationally, 76% of UK University staff were aged between 26 and 55 years; 80.7% of UK University staff were white, 2.4% were black, 7.3% were Asian, 1.8% were Mixed, and 1.4% “other.” 6.4% did not state their ethnicity (36). These figures are similar to our sample.

In 2017/18, there were 2,801,580 students attending UK Universities, with almost 84% being UK nationals, 5% EU nationals, and 11% from non-EU countries (38). Student ethnicity and age data was not available for non-UK nationals. For UK national students, 41% were aged 20 years or younger, 28% were aged between 21 and 24 years, 11% were aged between 25 and 29 years and 20% were aged 30 years or older. 76% of UK national students were white, 7% were black, 11% were Asian, 4% were mixed and 2% “other” (37). This is in line with our sample. Although the national data is from the 2017/18 academic year, the data suggests that our sample is representative of UK University students and staff members.

In addition, the question focused on exercise levels did not allow us to retrieve data on exercise type or duration and questions focused on green space did not ask participants on how much time they spent outside of their home. There is evidence that COVID-19 transmission mostly occurs indoors (65). It is a limitation of the study that we did not investigate whether those who spent more time exercising or accessing green space were less likely to be social isolating or have contracted COVID-19.

### Strengths of the Study

This is a timely survey, taken at the beginning of the upheaval of the outbreak and the lockdown, shortly after staff and students were sent home. It provides a unique insight in psychological distress, presenteeism and absenteeism, and their predictors, in a large, representative sample with both UK and international University staff and students. The substantial samples allowed us to explore interesting associations among the variables in the tertiary education sector that was heavily impacted by the COVID-19 outbreak. This study provides insights in the response of this group to a shared major social event and provides insights that so far were not explored. That can be considered a strength of the study.

### Implications for Public Health Interventions

The outcomes of this study suggest that there is scope to support staff and students with psychological distress to deal with that. However, the outcomes show as well that we could go a step further by supporting health promotion lifestyle interventions such as promoting exercise. Furthermore, there is scope to support vulnerable groups such as young female staff and students who have to combine care for young children with remote work and study to provide lenience with study and work deadlines, and to provide support for students and staff living with a disability. Reaching out to small, vulnerable groups such as non-binary gender groups, or BAME staff or students who are known to be more vulnerable to the virus, and enquire if they would need any help, might be warranted. Furthermore, Chinese students might be in need of support directed at them in a culturally adapted way.

### Implications for Further Research

This study shows that there is scope to explore vulnerability and resilience to a major social event inflicting on the work and study situation by a longitudinal design. As several of the strategies suggested above may have been (partly) implemented over time, the impact of that support might be explored in a longitudinal study. Also, more in-depth exploration of factors contributing to vulnerability and resilience would be needed.

## Conclusion

It is noteworthy that resilience occurred much more often in students than in staff, whereas psychological distress was much higher in students. This suggests that predictors of resilience may differ from psychological distress *per se*. In other words, a person may feel psychological distress and nevertheless be resilient. Hence, interventions to improve resilience should not only address psychological distress but may also address different factors that contribute to resilience, or aim at improving skills to deal with stressors.

## Data Availability Statement

The datasets presented in this article are not readily available because the participants did not consent to share their raw data. However, interested parties will be able to obtain data upon request as follows. Researchers can submit a research plan, which describes the background and methods of a proposed research question, and a request for specific data of the database used for this study to answer the research question. After approval of the research plan by the principal investigator and the University of York research governance board, a deidentified minimal dataset can be obtained. Information can be requested by contacting the principal investigator. Requests to access the datasets should be directed to CF-C christina.vanderfeltz-cornelis@york.ac.uk.

## Ethics Statement

The studies involving human participants were reviewed and approved by University of York Department of Health Sciences Research Governance Board. The patients/participants provided their written informed consent to participate in this study.

## Author Contributions

CV obtained funding and ethical approval for the study and wrote the study protocol. DV developed the survey, programmed it in Qualtrics, and extracted the data. CV, DV, VA, and EB designed the study, analysis plan, and analyzed the data. CV wrote the first draft. All authors contributed to the article and approved the final version.

## Conflict of Interest

The authors declare that the research was conducted in the absence of any commercial or financial relationships that could be construed as a potential conflict of interest.

## References

[1] Dolton P The Economics of the UK University System in the Time of Covid-19. London: National Institute of Economic and Social Research (2020).

[2] VOXEU The COVID-19 Pandemic Is Causing a Crisis in the UK Universities. (2020). Available online at: https://voxeu.org/article/covid-19-pandemic-causing-crisis-uk-universities (accessed July 27, 2020).

[3] McKie A Chinese Students in UK ‘Report Increased Racism and Discrimination’. Times Higher Education (2020). Available online at: https://www.timeshighereducation.com/news/chinese-students-uk-report-increased-racism-and-discrimination (accessed July 27, 2020).

[4] Guy J East Asian Student Assaulted in ‘Racist’ Coronavirus Attack in London. CNN (2020) Available online at: https://edition.cnn.com/2020/03/03/uk/coronavirus-assault-student-london-scli-intl-gbr/index.html (accessed July 27, 2020).

[5] Mercer D Coronavirus: Hate Crimes Against Chinese People Soar in UK During COVID-19 Crisis. Sky News (2020). Available online at: https://news.sky.com/story/coronavirus-hate-crimes-against-chinese-people-soar-in-uk-during-COVID-19-crisis-11979388 (accessed July 27, 2020).

[6] British Council HE Institutions Face Battle for Chinese Students as 39 per Cent of Applicants Unsure About Cancelling Study Plans. British Council Available online at: https://www.britishcouncil.org/contact/press/higher-education-chinese-students-covid-report (accessed July 27, 2020).

[7] Brackley J Institutions at Risk Due to Covid-19: A Tool Kit for Members and Negotiators. USS Briefs Available online at: https://medium.com/ussbriefs/institutions-most-at-risk-due-to-covid-19-a-tool-kit-for-members-and-negotiators-5829a7c2ae2d (accessed July 27, 2020).

[8] London Economics Impact of the COVID-19 Pandemic on University Finances. London: London Economics (2020).

[9] Aronsson G Gustafsson K Dallner M. Sick but yet at work. An empirical study of sickness presenteeism. J Epidem Comm Health. (2000) 54:502–9. 10.1136/jech.54.7.502 10846192 PMC1731716

[10] Dew K Keefe V Small K. Choosing to work when sick: workplace presenteeism. Soc Sci Med. (2005) 60:2273–82. 10.1016/j.socscimed.2004.10.022 15748675

[11] Burton NW Pransky G Conti DJ Chen C Edington DW. The association of medical conditions and presenteeism. J Occupat Environ Med. (2004) 46:S38–45. 10.1097/01.jom.0000126687.49652.44 15194894

[12] Lang SS Economists Coin New Word, ‘Presenteeism,’ To Describe Worker Slowdowns. (2004). Available online at: http://www.news.cornell.edu/stories/2004/04/new-word-job-health-problempresenteeism (accessed July 27, 2020).

[13] Hemp P. Presenteeism: at work – but out of it. Harvard Bus Rev. (2004) 82:49–58. 15559575

[14] Aronsson G Gustafsson K. Sickness presenteeism: prevalence, attendance pressure factors, and an outline of a model for research. J Occupat Environ Med. (2005) 47:9. 10.1097/01.jom.0000177219.75677.17 16155481

[15] Garrow V Presenteeism. A Review of Current Thinking. Brighton: Institute of Employment Studies (2016).

[16] Werner EE What can we learn about resilience from large scale longitudinal studies? In: Goldstein S Brooks RB editors. Handbook of Resilience in Children. New York, NY: Kluwer Academic/Plenum Publishers (2004). p. 87–102.

[17] Steinhardt MA Mamerow MM Brown SA Jolly CA. A resilience intervention in African American adults with type 2 diabetes: a pilot study of efficacy. Diabetes Educ. (2009) 35:274–84. 10.1177/0145721708329698 19204102 PMC3001398

[18] Werner E Through the Eyes of Innocents. New York, NY: Basic Books (2001).

[19] Konnikova M How People Learn to Become Resilient. The New Yorker (2016) Available online at: https://www.newyorker.com/science/maria-konnikova/the-secret-formula-for-resilience (accessed July 27, 2020).

[20] Avey JB Luthans F Smith RM Palmer NF. Impact of positive psychological capital on employee well-being over time. J Occup Health Psychol. (2010) 15:17–28. 10.1037/a0016998 20063956

[21] Werner EE Smith RS. Journeys From Childhood to Midlife: Risk, Resilience, and Recovery. New York, NY: Cornell University Press (2001). 10.1542/peds.114.2.492 15286237

[22] Likert R A Technique for the measurement of attitudes. Arch Psychol. (1932) 140:1–55.

[23] Jovancic N Likert Scale: How to Create Your Own Survey. (2019). Available online at: https://www.leadquizzes.com/blog/likert-scale/ (accessed March 9, 2020).

[24] Levenstein S Prantera C Varvo V Scribano ML Berto E Luzi C. Development of the perceived stress questionnaire: a new tool for psychosomatic research. J Psychosom Res. (1993) 37:19–32. 10.1016/0022-3999(93)90120-5 8421257

[25] Shahid A Wilkinson K Marcu S Shapiro CM STOP, THAT and One Hundred Other Sleep Scales. New York, NY: Springer-Verlag (2012). 10.1007/978-1-4419-9893-4

[26] Fliege H Rose M Arck P Walter OB Kocalevent R-D Weber C. The perceived stress questionnaire (PSQ) reconsidered: validation and reference values from different clinical and healthy adult samples. Psychosom Med. (2005) 67:78–88. 10.1097/01.psy.0000151491.80178.78 15673628

[27] Spitzer RL Kroenke K Williams JB Löwe B. A brief measure for assessing generalized anxiety disorder: the GAD-7. Arch Int Med. (2006) 166:1092–7. 10.1001/archinte.166.10.1092 16717171

[28] Kroenke K Spitzer RL Williams JB. The PHQ-9: validity of a brief depression severity measure. J Gen Inter Med. (2001) 16:606–13. 10.1046/j.1525-1497.2001.016009606.x 11556941 PMC1495268

[29] Kroenke K Spitzer RL Williams JB. The PHQ-15: validity of a new measure for evaluating the severity of somatic symptoms. Psychosom Med. (2002) 64:258–66. 10.1097/00006842-200203000-00008 11914441

[30] Hoedeman R Krol B Blankenstein N Koopmans PC Groothoff JW. Severe MUPS in a sick-listed population: a cross-sectional study on prevalence, recognition, psychiatric co-morbidity and impairment. BMC Pub Health. (2009) 9:440. 10.1186/1471-2458-9-440 19951415 PMC2793259

[31] van der Feltz-Cornelis CM Meeuwissen JA de Jong FJ Hoedeman R Elfeddali I. Randomised controlled trial of a psychiatric consultation model for treatment of common mental disorder in the occupational health setting. BMC Health Serv Res. (2007) 7:29. 10.1186/1472-6963-7-29 17326830 PMC1810250

[32] van Ravesteijn HJ Wittkampf K Lucassen P van de Lisdonk E van den Hoogen H van Weert WH. Detecting somatoform disorders in primary care with the PHQ-15. Ann Fam Med. (2009) 7:232–8. 10.1370/afm.985 19433840 PMC2682971

[33] Central Bureau of Statistics Netherlands Central Bureau of Statistics (CBS) List. Den Haag: Central Bureau of Statistics (2005).

[34] Bouwmans C Krol M Severens H Koopmanschap M Brouwer W Hakkaart-van Roijen L. The iMTA productivity cost questionnaire: a standardized instrument for measuring and valuing health-related productivity losses. Val Health. (2015) 18:753–8. 10.1016/j.jval.2015.05.009 26409601

[35] Cohen H What is Resilience? Available online at: https://psychcentral.com/lib/what-is-resilience/ (accessed May 6, 2020).

[36] HESA Higher Education Staff Statistics: UK. Cheltenham: HESA (2019).

[37] HESA Higher Education Student Statistics: UK - Student Numbers and Characteristics. Cheltenham: HESA (2019).

[38] Universities UK International International Facts and Figures 2019. London: Universities UK International (2019).

[39] Miller-Lewis LR Seale AK Sawyer MG Baghurst PA Hedley D. Resource factors for mental health resilience in early childhood: An analysis with multiple methodologies. Child Adolesc Psychiatry Ment Health. (2013) 7:6. 10.1186/1753-2000-7-6 23432929 PMC3598384

[40] Cosco TD Kaushal A Hardy R Richards M Kuh D Stafford M. Operationalising resilience in longitudinal studies: a systematic review of methodological approaches. J Epidemiol Community Health. (2017) 71:98–104. 10.1136/jech-2015-206980 27502781 PMC5256275

[41] Levinson DJ A conception of adult development. Am Psych. (1986) 41:3–13. 10.1037/0003-066X.41.1.3

[42] Löwe B Decker O Müller S Brähler E Schellberg D Herzog W. Validation and standardization of the generalized anxiety disorder screener (GAD-7) in the general population. Med Care. (2008) 45:266–74. 10.1097/MLR.0b013e318160d093 18388841

[43] Parkerson HA Thibodeau MA Brandt CP Zvolensky MJ Asmundson GJ. Cultural-based biases of the GAD-7. J Anxiety Dis. (2015) 31:38–42. 10.1016/j.janxdis.2015.01.005 25725310

[44] Kocalevent RD Hinz A Brähler E. Standardization of the depression screener patient health questionnaire (PHQ-9) in the general population. Gen Hosp Psychiat. (2013) 35:551–5. 10.1016/j.genhosppsych.2013.04.006 23664569

[45] Fu W Wang C Zou L Guo Y Lu Z Yan S. Psychological health, sleep quality, and coping styles to stress facing the COVID-19 in Wuhan, China. Translat Psychiat. (2020) 10:1–9. 10.1038/s41398-020-00913-3 32647160 PMC7347261

[46] Sartorao-Filho CI Rodrigues WCDLV de Castro RB Marcal AA Pavelqueires S Takano L Impact of COVID-19 Pandemic on mental health of medical students: a cross-sectional study using GAD-7 and PHQ-9 questionnaires. medRxiv [Preprint]. (2020). 10.1101/2020.06.24.20138925

[47] Pieh C Budimir S Probst T. The effect of age, gender, income, work, and physical activity on mental health during coronavirus disease (COVID-19) lockdown in Austria. J Psychosomat Res. (2020) 136:110186. 10.1016/j.jpsychores.2020.110186 32682159 PMC7832650

[48] Bergdahl J Bergdahl M Perceived stress in adults: prevalence and association of depression, anxiety and medication in a Swedish population. Stress and Health: J Internat Soc Investigat Stress. (2002) 1835–41. 10.1002/smi.946

[49] World Health Organization Adolescent Mental Health. Available online at: https://www.who.int/news-room/fact-sheets/detail/adolescent-mental-health (accessed July 27, 2020).

[50] Helbich M O'Connor RC Nieuwenhuijsen M Hagedoorn P. Greenery exposure and suicide mortality later in life: a longitudinal register-based case-control study. Environ Int. (2020) 143:105982. 10.1016/j.envint.2020.105982 32712421

[51] Kleinman A. Neurasthenia and depression: a study of somatization and culture in China. Cult Med Psychiatry. (1982) 6:117–90. 10.1007/BF00051427 7116909

[52] Ryder AG Chentsova-Dutton YE. Depression in cultural context:“Chinese somatization,” revisited. Psychiatr Clin N Am. (2012) 35:15–36. 10.1016/j.psc.2011.11.006 22370488

[53] Zhou X Dere J Zhu X Yao S Chentsova-Dutton YE Ryder AG. Anxiety symptom presentations in Han Chinese and Euro-Canadian outpatients: is distress always somatized in China? J Affect Disord. (2011) 135:111–4. 10.1016/j.jad.2011.06.049 21794924

[54] Kleinman A Kleinman J Remembering the cultural revolution: alienating pains and the pain of alienation/transformation. In: Lin TY Tsen WS Yeh E editors, Mental Health in Chinese Societies. Hong Kong: Oxford University Press (1995). p. 141–55.

[55] Kleinman A Anderson J Finkler K Frankenberg R Young A Social origins of distress and disease: depression, neurasthenia, and pain in modern China. Curr Anthropol. (1986) 24:499–509. 10.1086/203474

[56] Xu X Li XM Zhang J Wang W. Mental health-related stigma in China. Issues Ment Health Nurs. (2017) 39:126–34. 10.1080/01612840.2017.1368749 29053392

[57] Li W Moriyama M Cui Y Kazawa K Nakaya T Susanto T. Presenteeism among Chinese workers in Japan and its relationship with mental health and health-promoting lifestyles. Ind Health. (2020) 58:35–45. 10.2486/indhealth.2018-0201 31257231 PMC6997712

[58] Anderson E Shivakumar G. Effects of exercise and physical activity on anxiety. Front Psychiat. (2013) 4:27. 10.3389/fpsyt.2013.00027 23630504 PMC3632802

[59] Craft LL Perna F The benefits of exercise for the clinically depressed. Prim Care Companion J Clin Psychiat. (2004):104–11. 10.4088/PCC.v06n0301 PMC474733 15361924

[60] Avishai-Cohen H Zerach G Exposure to potentially traumatic events, posttraumatic stress symptoms, pain catastrophizing, and functional somatic symptoms among individuals with varied somatic symptoms: a moderated mediation model. J Interpers Violence. (2020) 2020:886260520912587 10.1177/0886260520912587 32326819

[61] van der Feltz-Cornelis CM van Dyck R The notion of somatization: an artefact of the conceptualization of body and mind. Psychother Psychosom. (1997) 66:117–27. 10.1159/000289121 9176904

[62] Hinkle DE Wiersma W Jurs SG Applied Statistics for the Behavioral Sciences. 5th ed Boston: Houghton Mifflin (2003).

[63] Carroll N Sadowski A Laila A The Impact of COVID-19 on health behavior, stress, financial and food security among middle to high income canadian families with young children. Nutrients. (2020) 12:2352 10.3390/nu12082352 PMC7468859 32784530

[64] Pierce M McManus S Jessop C John A Hotopf M Ford T Says who? The significance of sampling in mental health surveys during COVID-19. Lancet Psychiatry. (2020) 7:567–8. 10.1016/S2215-0366(20)30237-6 32502467 PMC7266586

[65] Qian H Miao T Liu L Zheng X Luo D Indoor transmission of SARS-CoV-2. medRxiv [Preprint]. (2020). 10.1101/2020.04.04.20053058 33131151

